# Measurement of plasma choline in acute coronary syndrome: importance of suitable sampling conditions for this assay

**DOI:** 10.1038/s41598-018-23009-x

**Published:** 2018-03-16

**Authors:** Ryunosuke Ohkawa, Makoto Kurano, Noboru Sakai, Tatsuya Kishimoto, Takahiro Nojiri, Koji Igarashi, Shigemi Hosogaya, Yukio Ozaki, Tomotaka Dohi, Katsumi Miyauchi, Hiroyuki Daida, Junken Aoki, Shigeo Okubo, Hitoshi Ikeda, Minoru Tozuka, Yutaka Yatomi

**Affiliations:** 1 Analytical Laboratory Chemistry, Graduate School of Health Care Sciences, Tokyo Medical and Dental University, Tokyo, Japan; 2 Department of Clinical Laboratory, The University of Tokyo Hospital, Tokyo, Japan; 3 Department of Clinical Laboratory Medicine, Graduate School of Medicine, The University of Tokyo, Tokyo, Japan; 4 Diagnostics R&D Division, Alfresa Pharma Corporation, Osaka, Japan; 5 Bioscience Division, Research and Development Management Department, TOSOH Corporation, Kanagawa, Japan; 6 Department of Medical Technology, School of Health Sciences, Tokyo University of Technology, Tokyo, Japan; 7 Department of Clinical and Laboratory Medicine, Faculty of Medicine, University of Yamanashi, Yamanashi, Japan; 8 Department of Cardiovascular Medicine, Juntendo University School of Medicine, Tokyo, Japan; 9 Department of Molecular and Cellular Biochemistry, Graduate School of Pharmaceutical Sciences, Tohoku University, Miyagi, Japan; 10 PRESTO, Japan Science and Technology Corporation, Tokyo, Japan; 11 Department of Clinical Laboratory Medicine, Faculty of Health Science Technology, Bunkyo Gakuin University, Tokyo, Japan

## Abstract

Blood choline has been proposed as a predictor of acute coronary syndrome (ACS), however different testing procedures might affect the choline concentration because the lysophospholipase D activity of autotaxin (ATX) can convert lysophosphatidylcholine to lysophosphatidic acid (LPA) and choline in human blood. Although the influences of ATX on LPA levels are well known *in vivo* and *in vitro*, those on choline have not been elucidated. Therefore, we established suitable sampling conditions and evaluated the usefulness of plasma choline concentrations as a biomarker for ACS. Serum LPA and choline concentrations dramatically increased after incubation depending on the presence of ATX, while their concentrations in plasma under several conditions were differently modulated. Plasma choline levels in genetically modified mice and healthy human subjects, however, were not influenced by the ATX level *in vivo*, while the plasma LPA concentrations were associated with ATX. With strict sample preparation, the plasma choline levels did not increase, but actually decreased in ACS patients. Our study revealed that ATX increased the choline concentrations after blood sampling but was not correlated with the choline concentrations *in vivo*; therefore, strict sample preparation will be necessary to investigate the possible use of choline as a biomarker.

## Introduction

Choline is quaternary ammonium ion with an N, N, N-trimethylethanolammonium cation structure that is required for components of the cell membrane, such as choline-containing phospholipids (phosphatidyl choline and sphingomyelin)^1^, and for neurotransmission as a precursor of acetylcholine^2^. Choline is also important for folate dependent one-carbon metabolism^3^. Choline is converted to betaine by oxidation, and betaine serves as a methyl donor to produce methionine by the remethylation of homocysteine, which has been proposed as a possible risk factor for coronary artery disease (CAD)^4^. Since choline has potentially important roles in overall homeostasis, as described above, many clinical studies have investigated the importance of choline as a biomarker for several human diseases^5–13^. Among them, the association between choline and CAD has been well investigated^9–13^, because choline can be produced by phospholipase D (EC 3.1.4.4), which is reportedly associated with myocardial infarction^10,14^. Several studies and international guidelines have indicated that an increased level of whole-blood choline has been suggested as a predictor of CAD^11–13,15–17^. In addition to whole-blood choline, measurements of choline in plasma and serum have been proposed to be predictive for acute coronary syndrome (ACS)^13,15,18^. Contrary to whole-blood choline, however, the association between plasma choline levels and ACS remains controversial^12,13,19^, and the diagnostic usefulness of plasma and serum choline levels has not been fully elucidated. One of the possible reasons for these discrepancies might be that the sampling method for measuring choline might affect the results; there are some concerns that differences in sampling, such as the type of tube used to collect the blood sample, the procedures used to prepare the plasma and serum samples, and the physiological conditions of the subjects, might affect the choline concentrations.

At present, the effects of different sample preparations on the concentrations of choline in clinical samples have not been fully clarified. For example, one candidate that might affect the choline level during sample preparation is autotaxin (ATX)(EC 3.1.4.39). ATX was originally characterized as a secreted enzyme with an autocrine motility factor from the conditioned medium of A2058 human melanoma cells^20^, and this enzyme is identical to lysophospholipase D (LysoPLD), which converts lysophosphatidylcholine (LPC) to a bioactive lipid mediator, lysophosphatidic acid (LPA), and choline^21,22^. In a previous study, we demonstrated that the LPA concentrations were increased when serum samples were left to stand at room temperature or at 37 °C, possibly because of the bioactivities of ATX^23^. The influences of ATX on LPA levels were also observed *in vivo* as well as *in vitro*; the levels of both ATX and LPA in ATX heterozygote mice were half the levels observed in wild-type animals^24,25^, and our previous study showed a strong correlation between serum ATX activity and the plasma LPA concentration in healthy human subjects^26^. Thus, ATX is deeply involved in the production of LPA both *in vitro* and *in vivo*. On the other hand, the relationship between the ATX level and choline, the other product of ATX, has not yet been elucidated.

Therefore, in this study, we investigated the stability of choline concentrations during sample preparation and the impact of ATX on the homeostasis of choline both *in vitro* and *in vivo*. Moreover, using the suitable sample handling established in the present study, we measured the plasma choline levels in both healthy subjects and patients who had undergone coronary angiography to investigate the possibility of its use as a biomarker.

## Results

### Validation of an enzymatic choline assay

We first validated our current choline assay. The calibration curve was shown in Fig S1a. The regression line for this assay was linear up to at least 123.6 μmol/L (Fig S1b). The minimum detection limit was 1.8 μmol/L. The within-run coefficient variation (n = 10) was 0.4% at a mean choline concentration of 10.5 μmol/L. The between-run coefficient variation (n = 10) was 2.9% at the mean choline concentration of 44.0 μmol/L.

### Variations in choline and LPA levels under various conditions

**Figure 1:**
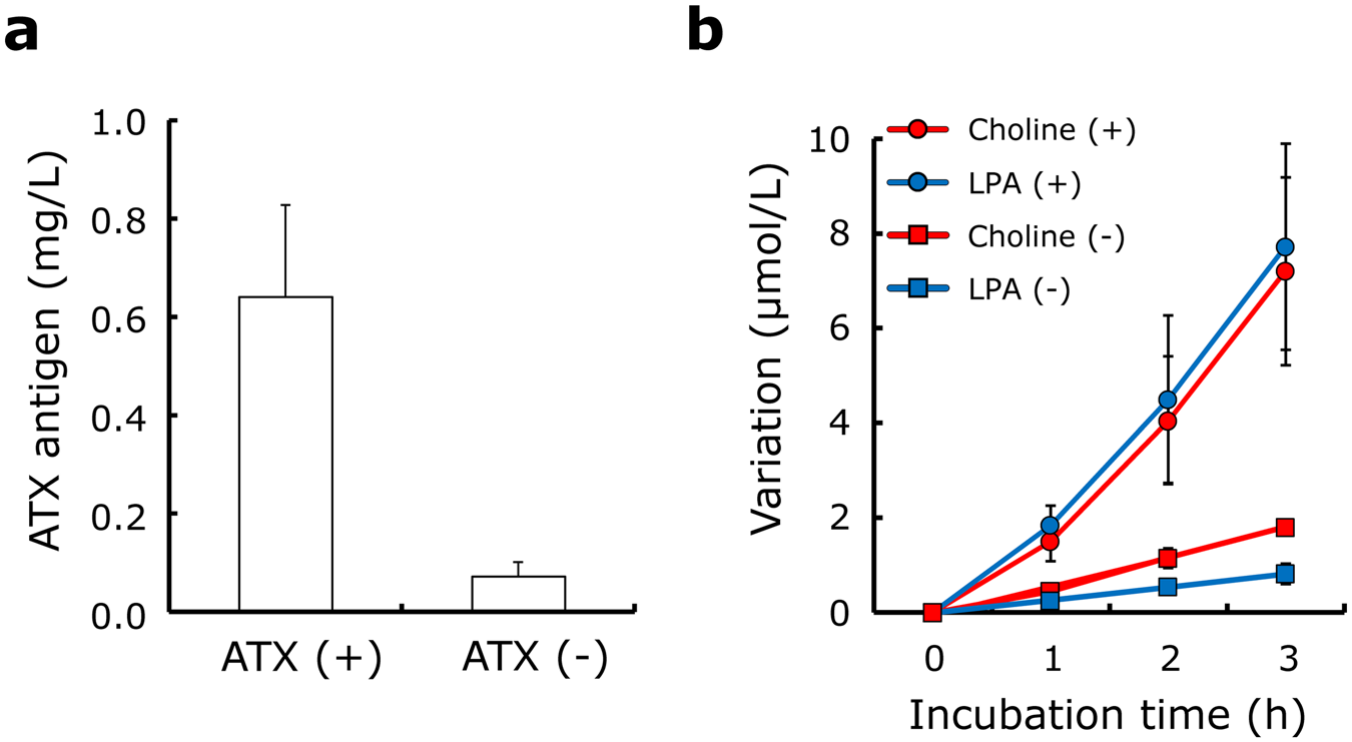
Variations in serum choline and LPA levels after incubation. ATX antigen levels were measured in normal human serum (ATX (+)) and ATX-depleted serum (ATX (−)) prepared using biotin-anti-ATX antibody and streptavidin-bound magnetic beads (**a**). Normal human serum (+)(circle) and ATX-depleted serum (−)(square) were incubated at 37 °C for 1 to 4 hours, and the levels of choline (red) and LPA (blue) were determined (**b**). Results are given as the mean ± SD (n = 2).

First, ATX-depleted sera were prepared from normal human serum using anti-ATX antibody-bound magnetic beads. Using this immune precipitation, the ATX antigen level in normal human serum was reduced by 88.5% from 0.64 ± 0.19 mg/L to 0.07 ± 0.03 mg/L (Fig. 1a). To investigate whether ATX produces both choline and LPA at the same time in serum, normal and ATX-depleted sera were incubated at 37 °C for 1–3 hours. The choline and LPA concentrations in untreated serum after incubation for 3 hours were increased by 7.7 ± 2.2 μmol/L and 7.2 ± 1.8 μmol/L, respectively, while the depletion of ATX attenuated the increases of both choline and LPA (Fig. 1b).

**Figure 2:**
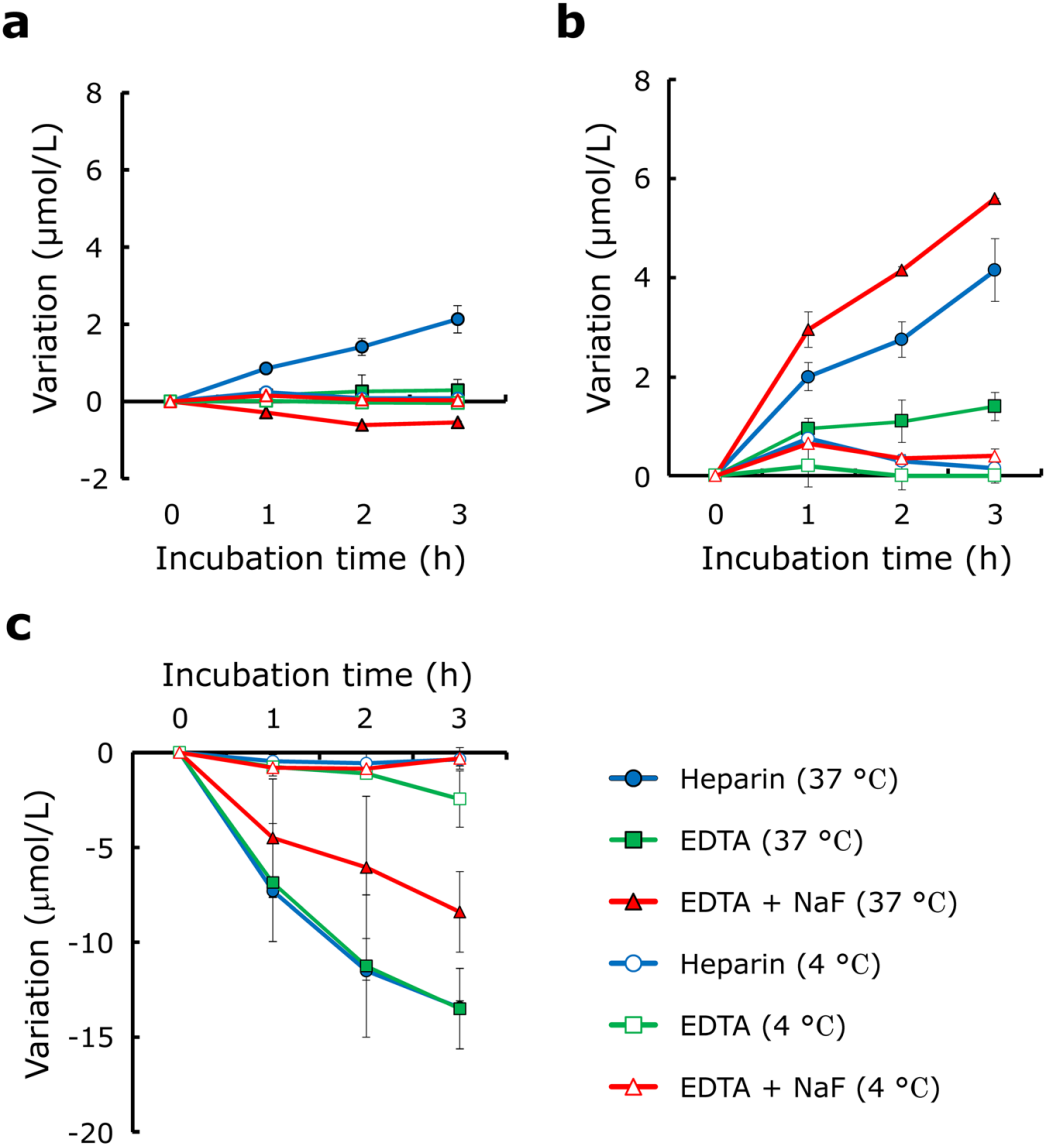
Variations in plasma choline and LPA levels obtained from whole blood with the addition of various anticoagulants. Whole blood obtained from healthy human subjects was mixed with heparin (blue), EDTA-2K (EDTA)(green), or EDTA-2K and NaF (EDTA + NaF)(red), then incubated at 37 °C (closed mark) or 4 °C (open mark) for 1 to 4 hours. After the plasma samples were collected by centrifugation at 4 °C, the concentrations of LPA (**a**) and choline (**b**) were measured in each plasma sample. (**c**) Eighty-micromolar choline chloride was added to whole blood with heparin, EDTA-2K (EDTA), or EDTA-2K and NaF (EDTA + NaF), then incubated at 37 °C or 4 °C for 1 to 4 hours. After the plasma samples were collected by centrifugation at 4 °C, the concentrations of choline were measured in each plasma sample. Results are given as the mean ± SD (n = 2).

Next, to investigate whether the method of plasma preparation affects the choline levels, whole blood was mixed with 1 mg/mL of ethylenediamine tetraacetic acid dipotassium salt 2H_2_O (EDTA-2K), EDTA-2K + sodium fluoride (NaF), or heparin and incubated at 37 °C or 4 °C for 1 to 3 hours; the plasma choline and LPA levels were then compared among the plasma samples. When whole blood plus heparin was incubated at 37 °C for 3 hours, both the plasma LPA and choline concentrations were increased by 2.1 ± 0.2 μmol/L and 3.4 ± 0.7 μmol/L (Fig. 2a,b), respectively. Regarding whole blood in the presence of EDTA-2K, the levels of plasma choline were increased up to 1.6 ± 0.2 μmol/L after 3 hours of incubation, while the plasma LPA concentrations were increased by only 0.3 ± 0.0 μmol/L (Fig. 2a,b). Moreover, when whole blood was prepared with EDTA + NaF, the plasma choline levels were increased by 5.8 ± 0.1 μmol/L, while the plasma LPA concentrations were decreased by 0.5 ± 0.1 μmol/L (Fig. 2a,b). The plasma choline and LPA concentrations were not changed when each whole blood sample was incubated at 4 °C. Furthermore, to investigate whether excessive choline is regulated by blood cells, 80 μmol/L of choline chloride was added to each whole blood sample before incubation. Consequently, the plasma choline levels in whole blood with heparin, EDTA-2K, and EDTA-2K + NaF were respectively decreased by 13.5 ± 5.2 μmol/L, 13.5 ± 5.4 μmol/L, and 8.4 ± 2.1 μmol/L after incubation at 37 °C for 3 hours, whereas these decreases were suppressed when incubated at 4 °C (Fig. 2c). Since the choline level was increased regardless of the tube type when the samples were not kept at 4 °C, blood samples should be immediately placed on ice until the preparation of the plasma sample by centrifugation to measure choline levels in healthy subjects and patients, as described below.

### Levels of plasma choline and LPA in conditional ATX knock-out or overexpression mice

**Figure 3:**
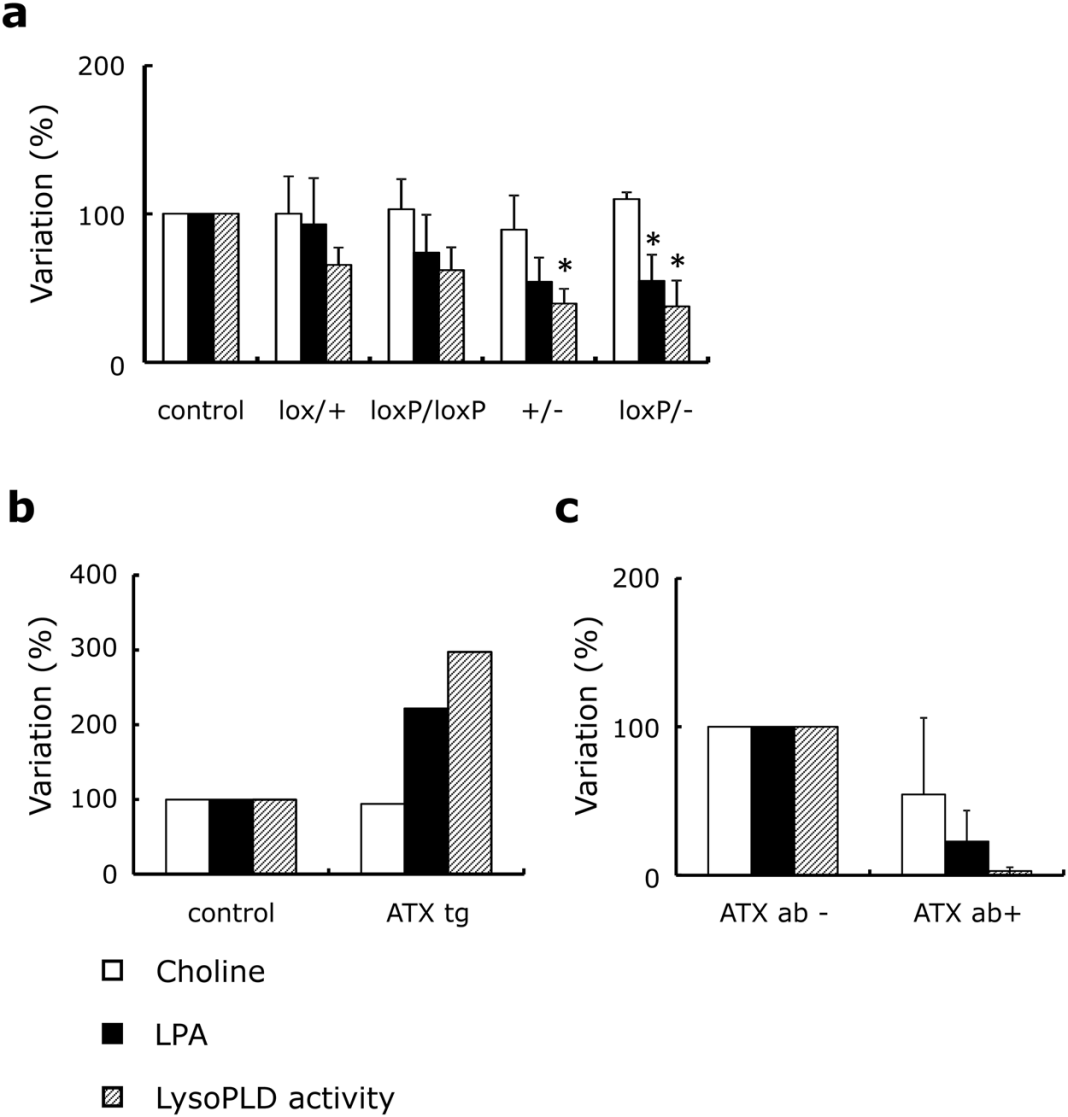
Plasma choline, LPA and serum LysoPLD activity in ATX knock out or overexpression mice. Plasma and serum samples were collected from each type of genetically modified mouse: control (n = 7), lox/ + (n = 5), loxP/loxP (n = 9), +/− (n = 2), and loxP/− (n = 4) mice (**a**), control or ATX transgenic (ATX tg) mice (n = 1, each) (**b**), or anti-ATX antibody treated (ATX ab+) or untreated (ATX ab−) mice (n = 3, each) (**c**). The concentrations of choline and LPA in the plasma and the activities of LysoPLD in the serum were measured. Results are given as the mean ± SD of the percentage of the value in the control. *P < 0.05 versus control.

Since choline and LPA, both of which are mainly produced by ATX from LPC, were not equally regulated, we investigated the effect of the ATX levels on the choline and LPA levels *in vivo* using ATX knock-out or overexpression mice. As indicated in Fig. 3a, the ATX activity tended to be reduced depending on each mouse genotype (lox/+ and loxP/loxP)(*P* = 0.190 and 0.063, respectively) and were significantly lower in+/− and loxP/− mice than in control mice (*P* = 0.036 and 0.020, respectively). Consistent with these results, LPA concentrations tended to be lower in loxP/loxP and +/− mice (*P* = 0.094 and 0.145, respectively) and were significantly lower in loxP/− mice than in control mice (*P* = 0.020). However, the plasma choline levels did not differ among the groups (*P* = 0.775). The percentages of LysoPLD activities in lox/+, loxP/loxP, +/−, and loxP/- mice relative to those in wild-type mice were 65.6%, 62.0%, 39.6%, and 37.6%, respectively. Concordant with the LysoPLD activities, the percentages of the LPA level were 93.0%, 74.0%, 54.3%, and 54.9%, respectively. Moreover, the percentages of LysoPLD activity and LPA in ATX transgenic mice, relative to those in wild-type mice, were increased by 297.5% and 222%, respectively, whereas the plasma choline level was decreased by only 5.9% (Fig. 3b). Treatment with anti-ATX antibody reduced the ATX activity and the LPA level in serum by 97.2% and 77.2%, while the plasma choline level was not modulated (Fig. 3c).

### Correlation of plasma choline levels with ATX-related metabolites and distribution of plasma choline concentrations in healthy subjects

**Figure 4:**
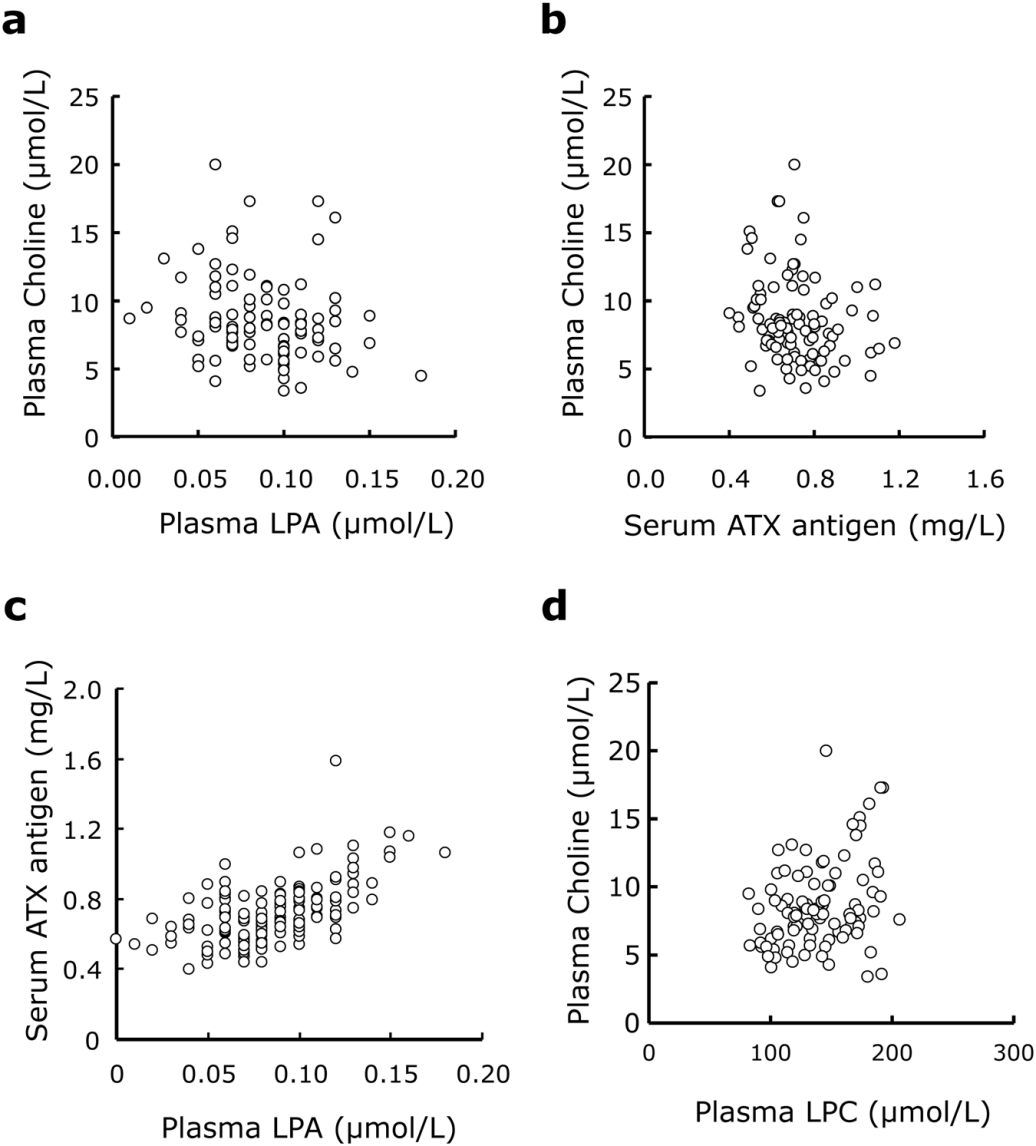
Correlations between plasma choline and ATX-related metabolites. Plasma choline, LPA, and LPC and serum ATX antigen levels were measured in healthy human subjects (n = 98). The correlations between plasma choline and plasma LPA (**a**), serum ATX antigen (**b**), and plasma LPC (**d**) and between serum ATX antigen and plasma LPA (**c**) were then analyzed.

We next compared the correlation of plasma choline with serum ATX antigen or plasma LPA concentrations in healthy human samples (n = 98). The plasma choline concentrations were weakly but significantly correlated negatively with both the plasma LPA (r = −0.232, *P* = 0.002) and serum ATX antigen levels (r = −0.235, *P* = 0.020) (Fig. 4a,b), while the plasma LPA level showed a significantly positive correlation with the serum ATX antigen level (r = 0.524, *P* < 0.001) (Fig. 4c). Regarding LPC, a precursor of LPA, the plasma choline concentration was correlated with LPC (r = 0.258, *P* = 0.010) (Fig. 4d).

In healthy subjects (n = 98), the mean ± SD of the plasma choline concentration was 9.6 ± 3.5 μmol/L. Plasma choline was significantly higher in men (10.3 ± 3.8 μmol/L) than in women (8.1 ± 2.2 μmol/L) (*P* < 0.001), while we previously showed that the plasma LPA and ATX antigen levels were higher among women than among men^26^. Table 1 shows the correlation between plasma choline and other parameters. The plasma choline concentrations were significantly and positively correlated with age, body mass index (BMI), LPC, urea nitrogen, creatinine, uric acid, aspartate aminotransferase, alkaline phosphatase, gamma-glutamyl transferase, hemoglobin, hematocrit, sodium and potassium and were significantly and negatively correlated with ATX activity and antigen and chloride.

**Table 1:** Correlation between plasma choline level and various parameters.

	*R*	*P*
Phospholipids metabolite-related parameters		
Autotaxin activity	−0.303	0.002
Autotaxin antigen	−0.235	0.020
Lysophosphatidic acid	−0.232	0.021
Lysophosphatidylcholine	0.258	0.010
Phosphatidylcholine	−0.010	0.928
Sphingomyelin	−0.102	0.353
Homocysteine	0.157	0.122
*Chemistry*		
High-density lipoprotein cholesterol	−0.066	0.520
Low-density lipoprotein cholesterol	0.014	0.887
Triglyceride	0.038	0.710
Urea nitrogen	0.234	0.020
Creatinine	0.293	0.003
Uric acid	0.408	<0.001
Aspartate aminotransferase	0.215	0.034
Alanine aminotransferase	0.170	0.094
Alkaline phosphatase	0.208	0.039
Gamma-glutamyl transferase	0.260	0.010
Sodium	0.258	0.010
Potassium	0.200	0.049
Chloride	−0.123	0.229
*Blood cell-related parameters*		
White blood cell count	0.052	0.611
Red blood cell count	0.133	0.192
Hemoglobin	0.233	0.021
Hematocrit	0.206	0.042
Platelet count	0.031	0.763

The number of samples examined ranged from 79 to 98.

### Choline and LPA levels under fasting and fed states

**Figure 5:**
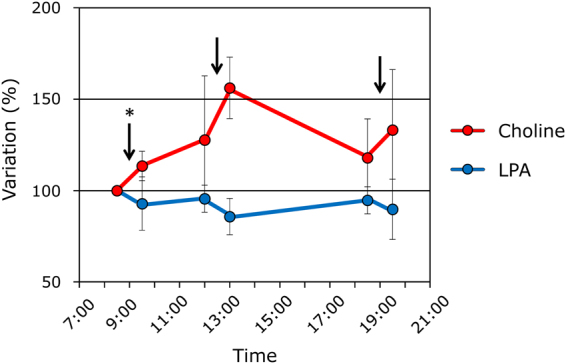
Plasma choline and LPA levels under fasting and fed states. The plasma choline (red) and LPA (blue) levels before and after meals (n = 5) were determined. The data show the percentage change from the value measured before the first meal (mean ± SD). The arrows indicate food intake. *P < 0.05, differences between before and after meals.

We next investigated the effect of meal consumption on the levels of choline and LPA. To analyze the modulation of choline and LPA by meal intake, we obtained blood samples serially before and at 30 min after each meal in 5 healthy subjects, who consumed 3 ordinary meals per day, and compared the dynamism of the plasma choline and LPA levels. As shown in Fig. 5, the plasma choline concentrations were significantly higher after first food intake (*P* = 0.042) and tended to be increased after the second (*P* = 0.080) and third (*P* = 0.068) food intakes, while no variation in the LPA levels was observed.

### Association of plasma choline concentration with cardiovascular events

**Figure 6:**
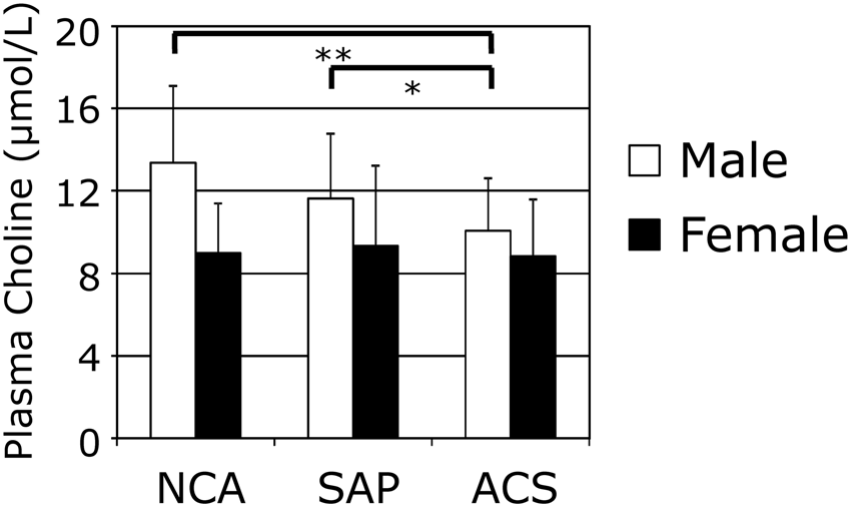
Association between plasma choline level and ACS. The concentrations of plasma choline in patients with NCA, SAP, or ACS are shown. The open bars represent the plasma choline levels in male subjects and the closed bars represent the levels in female subjects. Data are the mean ± SD; *P < 0.05, **P < 0.01.

We investigated the association between the plasma choline level and CAD. The basic characteristics of the three groups (normal coronary arteries (NCA), stable angina pectoris (SAP) and ACS) according to diagnosis were shown in a previous study^27^. The plasma choline level was significantly higher in men than in women (11.5 ± 3.3 vs. 9.0 ± 3.0 μmol/L, *P* < 0.001) as was the case in healthy subjects. In men, the plasma choline concentration was significantly decreased in the group with ACS (10.1 ± 2.6 μmol/L), compared with the NCA (13.3 ± 3.7 μmol/L, *P* = 0.003) and SAP (11.6 ± 3.1 μmol/L, *P* = 0.032) groups (Fig. 6), whereas no differences among the groups were observed in women. Therefore, we analyzed the association between the plasma choline levels and ACS-related factors in further detail in a men-only group. When all the male patients who had undergone coronary angiography (n = 110) were divided into four equal groups based on their plasma choline values, trends toward significant associations between an increase in the choline quartile and the red blood cell count, high sensitive C-reactive protein (hsCRP) level, and number of ACS subjects were observed (Table 2). Although, the troponin T levels in ACS group were higher than those in NCA and SAP groups as we described previously^27^, the significant correlation between plasma choline and troponin T was not observed. Finally, to further confirm the possible usefulness of plasma choline as a biomarker for the diagnosis of ACS, we constructed a logistic regression analysis examining male subjects only. When explanatory variables (age, BMI, current smoking, diabetes, red blood cell count, high-density lipoprotein cholesterol (HDL-C), low-density lipoprotein cholesterol (LDL-C), triglyceride, hsCRP, choline quartile and LPA tertile) were introduced into a stepwise multivariate model, only hsCRP (*P* = 0.047), choline quartile (*P* = 0.002) and LPA tertile (*P* = 0.004) were selected as significant predictors of ACS after adjustment (Table 3).

**Table 2:** Association between plasma choline quartile and risk factors for ACS.

**Characteristic**		**All Participants(n = 110)**	**Plasma choline concentration**				***P*** **value for trend**
			Quartile 1 (n = 28)	Quartile 2 (n = 27)	Quartile 3 (n = 27)	Quartile 4 (n = 28)	
Plasma Choline (μmol/L)		6.2–24.2	6.2–9.1	9.2–11.2	11.3–13.3	13.4–24.2	
Age							0.371
(year)	Median	65	68	62	67	67	
	Interquartile	57–73	54–73	55–73	58–76	61–71	
Body mass index							0.721
	Median	24.3	23.75	24.3	23.8	24.7	
	Interquartile	21.7–26.3	22.1–26.3	22.0–27.5	21.6–25.7	22.5–26.1	
Systolic blood pressure							0.410
(mm Hg)	Median	135	136	135	134	137	
	Interquartile	117–152	109–155	121–149	122–151	120–158	
Diastolic blood pressure							0.061
(mm Hg)	Median	76	78	78	70	72	
	Interquartile	65–84	68–87	67–88	65–80	63–79	
White blood cell counts							0.710
(10^3^ cell/μL)	Median	5.9	6.2	5.4	5.9	6.2	
	Interquartile	5.0–7.3	5.0–7.3	5.1–6.4	5.0–7.6	5.0–7.3	
Red blood cell counts							0.005
(10^6^ cell/μL)	Median	4.54	4.68	4.62	4.43	4.35	
	Interquartile	4.24–4.89	4.47–4.92	4.35–4.87	4.14–4.73	4.03–4.80	
Creatinine							0.432
(mg/dL)	Median	0.81	0.80	0.77	0.82	0.85	
	Interquartile	0.72–0.91	0.73–0.87	0.72–0.88	0.73–0.94	0.74–0.98	
High sensitive C-reactive protein							0.027
(mg/dL)	Median	0.05	0.05	0.04	0.05	0.06	
	Interquartile	0.02–0.15	0.03–0.07	0.01–0.14	0.02–0.14	0.02–0.45	
Troponin T							0.532
(pg/mL)	Median	5.2	5.1	6.8	4.4	6.9	
	Interquartile	3.0–25.0	3.1–24.6	3.1–19.9	2.9–9.2	3.9–81.3	
High-density lipoprotein cholesterol							0.847
(mg/dL)	Median	43	43	45	43	42	
	Interquartile	38–51	38–50	40–52	38–55	39–49	
Low-density lipoprotein cholesterol							0.080
(mg/dL)	Median	106	116	106	102	107	
	Interquartile	90–120	98–132	88–115	93–116	81–120	
Triglyceride							0.867
(mg/dL)	Median	118	124.5	118	108	118	
	Interquartile	85–167	86–170	80–158	98–159	85–183	
Angiographic degree of CAD							
1-vessel disease	Number		16	14	12	8	0.028
2-vessel disease	Number		5	8	10	6	0.634
3-vessel disease	Number		4	1	2	4	0.890
ACS/nonACS							0.01
	Number	29/81	12/16	7/20	6/21	4/24	6

**Table 3:** Multivariate logistic regression model for the prediction of acute coronary syndrome.

	Multivariate analysis OR (95% CI)	*P*
High sensitive C-reactive protein	2.811 (1.018–7.763)	0.047
Choline quartile, increment	0.432 (0.259–0.72)	0.002
Lysophosphatidic acid tertile, increment	2.865 (1.407–5.835)	0.004
Current smoking, yes	2.865 (1.407–5.835)	
Age	Not selected	
Body mass index	Not selected	
Diabetes, yes	Not selected	
Red blood cell count	Not selected	
High-density lipoprotein cholesterol	Not selected	
Low-density lipoprotein cholesterol	Not selected	
Triglyceride	Not selected	

## Discussion

ATX is an important enzyme determining LPA level in circulation and theoretically produces equal molar amounts of choline. However, under the condition where whole blood was incubated, these two substances showed different kinetics; the present study showed that the serum LPA and choline concentrations dramatically increased after incubation depending on the presence of ATX (Fig. 1), while the plasma LPA and choline concentrations were differently modulated when the samples were incubated under several conditions; in whole blood plus EDTA-2K, the increase in the plasma LPA level was suppressed by the inactivation of ATX with a chelating agent, while no remarkable modulation of the plasma choline levels was observed. In relation to the variations in the plasma choline level, the degree of increase in plasma obtained from whole blood prepared with heparin was higher than that in samples prepared with EDTA. These differences in increased plasma choline levels between EDTA and heparin are consistent with those of previous studies^19,28^. The difference in the choline kinetics between serum and whole blood indicates the presence of other factors from blood cells. Regarding the catabolism of choline, excessively added choline decreased in a time-dependent manner in whole blood samples (Fig. 2c). Although our study did not elucidate the exact underlying mechanism, considering that erythrocytes possess a choline influx-transporter, the choline might have been absorbed by the erythrocytes. Erythrocytes also have a choline transporter that regulates both the influx and efflux of choline^29^, and this choline transporter was inhibited by the blockage of Na+/K+ATPase (EC 3.6.1.3)^30^. This speculation was concordant with the results shown in Fig. 2c; the influx of choline into blood cells might be suppressed by NaF, which inhibits enolase resulting in a rapid decrease in the erythrocyte ATP concentration. In addition, the correlation between plasma choline levels and hemoglobin concentrations and hematocrit levels as well as the gender difference (higher in men) in healthy human subjects suggests the regulation of plasma choline by erythrocytes *in vivo*. Regarding the association between the plasma choline levels and ATX *in vivo*, while ATX was at least partly involved in the homeostasis of choline in the *in vitro* experiments, the results from the animal experiments with genetically modified mice revealed that the regulation of the circulating choline level was not influenced by the ATX level *in vivo*. Concordant with these results, in healthy human subjects, the plasma choline level was not correlated with ATX, while the plasma LPA level was positively correlated with ATX. Taken together, our results suggest that both ATX-dependent and ATX-independent pathways are involved in the regulation of plasma choline levels, with the later pathway preferred *in vivo*. However, the former pathway cannot be ignored when performing laboratory medicine tests, since the choline level changes according to the tube type and handling procedure in the presence of ATX as well as the transporters on blood cells *in vitro*. Therefore, samples should be kept at a low temperature at all times during processing, from blood collection until measurement. To our knowledge, this is the first study to reveal the impact of ATX on the plasma choline level.

Regarding the timing of blood sampling, it might be desirable to consider the influence of meals. Choline is abundant in foods, and the dietary intake of choline is necessary to obtain materials for cell components and others. Actually, choline deficiency causes various problems including hepatic steatosis^31^, muscle damage^32^, and lymphocyte apoptosis^33^. We investigated the influence of meals on the plasma choline and LPA levels and the diurnal variations of these concentrations. Our data indicated that blood collection should ideally be performed before meals to avoid transient increases in choline.

From analytical and preanalytical aspects, our study revealed that the measurement of serum choline levels is not appropriate because of its *in vitro* production by ATX. Considering the variations in plasma and serum choline levels, measuring whole-blood choline levels seems to be more suitable for reflecting the activity of released phospholipase. However, a mass spectrometry assay, which has been performed for the assessment of whole-blood choline levels in many studies, is difficult to introduce into laboratory testing because of the initial cost and reproducibility concerns. Therefore, the measurement of plasma choline using samples prepared with anti-coagulants, such as EDTA, on ice using an automatable enzymatic assay would be optimal for the clinical introduction of choline as a biomarker for human diseases.

Regarding the clinical use of choline as a biomarker for ACS, the fact that the activations of phospholipase A_2_ (EC 3.1.1.4) and phospholipase D^15^ in ischemic heart tissue lead to the release of choline into plasma provides a plausible explanation for why the increased choline level in plasma is similar to that in whole blood. Actually, regarding the use of plasma choline as a predictive marker, some studies have demonstrated the predictive value of increased plasma choline levels in patients with ACS^13,15,16^. However, Body *et al*. suggested that the plasma choline levels did not help to predict a diagnosis of acute myocardial infarction (AMI) (odds ratio, 1.00; 95% confidence interval, 0.91–1.10; *P* = 0.98)^12^. Unexpectedly, our study also showed that the plasma choline concentrations in the ACS group were rather lower than those in the non-ACS group, although the difference was not large. Several studies support the results from the present study; Danne *et al*. demonstrated that the whole-blood choline level in patients with AMI was increased to about 50–100 μmol/L, whereas the plasma choline remained low (about 10–15 μmol/L)^34^. More recently, Storm *et al*. suggested that the plasma choline levels decreased 6–12 hours after the return of spontaneous circulation and reached a subnormal concentration, with a median of 4.0 μmol/L^35^. Until now, no reports have described the use of prepared samples in which the blood collection tubes were kept on ice prior to centrifugation. The reason why the plasma choline levels were lower in our study than in other studies is likely due to the difference in the sample procedure before analysis. Regardless, even with the rigorous sample preparation protocol used in this study, the plasma choline levels were not increased, but rather decreased in the subjects with ACS, although blood sampling was not always performed under fasting conditions in the present study.

In summary, the present study revealed that although ATX did not affect choline levels *in vivo*, ATX could increase choline levels *in vitro*, suggesting the need for strict sample preparation. We also observed that the plasma choline levels were decreased in plasma samples suitably prepared from patients with ACS. Further studies to investigate the possible usefulness of the plasma choline level using this assay and optimized procedures are expected to be performed.

## Materials and Methods

### Ethics statement and guidelines compliance

All human samples were collected with informed and written consent. The Ethics Review Committee at Juntendo University Hospital approved the study (number: 291). This study was also approved by the institutional review boards of both the University of Tokyo (number: 2602 and 11158). and Juntendo University School of Medicine. All the animal experiments were conducted in accordance with the guidelines and regulations for Animal Care and were approved by the animal committee of Tohoku University (2013PhA-034). All methods were performed in accordance with the relevant guidelines and regulations.

### Chemicals

Unless otherwise stated, all the reagents were purchased from Wako Pure Chemical Industries (Osaka, Japan). Choline oxidase (EC 1.1.3.17), sphingomyelinase (EC 3.14.1.2) and lysophospholipase (EC 3.1.1.5) were obtained from Asahi Kasei Pharma (Tokyo, Japan). Peroxidase (EC 1.11.1.7) was purchased from Toyobo (Osaka, Japan). N-ethyl-N-(2-hydroxy-3-sulfopropyl)-3-methylaniline sodium salt (TOOS) was obtained from Dojindo Laboratories (Kumamoto, Japan). 4-Aminoantipyrine (4-AA) was obtained from Kishida Chemical (Osaka, Japan).

### Samples

The samples used to examine the basic profiles of choline were collected from the antecubital vein of healthy adult volunteers. The serum and plasma samples used to determine the reference ranges for choline were residual samples from those obtained for laboratory analyses (for medical checkups). These healthy subjects (n = 98) were defined as those who did not receive any medical treatment at the sampling. To prepare the plasma samples, the whole blood samples were collected into vacuum tubes containing 3 mg/mL of EDTA-2K and then mixed with 10% v/v of citrate-theophylline-adenosine-dipyridamole (CTAD)^36^ (BD Biosciences, Tokyo, Japan), as previously described^23^. The tubes were immediately placed in an ice/water bath, followed by 15 min of incubation. The anticoagulated samples were centrifuged at 2500 × *g* for 30 min to separate the plasma. As soon as centrifugation was completed, the plasma supernatant was carefully collected to avoid contamination with cell components. To obtain the serum samples, whole blood specimens were directly placed into a glass tube and left for 15 min at room temperature to allow blood clot formation; then, the serum was separated by centrifugation at 1500 × *g* for 5 min. The samples from patients who had undergone coronary angiography were obtained as previously described^27^. Briefly, subjects who underwent coronary angiography at Juntendo University Hospital (J-Bacchus trial) between July and December 2009 were enrolled according to the entry criteria (no previous examination by coronary angiography, no history of coronary intervention or coronary artery bypass grafting, and having precisely evaluable coronary trees). This is a prospective cross-sectional study registered in the UMIN protocol registration system (#UMIN000002103). One hundred fifty eight patients were initially screened for this study and excluded as follows: 1) maintenance dialysis (n = 5), 2) diabetes treated with insulin (n = 4), and 3) acute or chronic infectious (n = 5) or 4) neoplastic (n = 3) diseases. Patients without significant stenosis were placed in NCA group (n = 32; male/female, 20/12), whereas those with significant stenosis were placed in an ACS group (n = 38; male/female, 29/9) or SAP group (n = 71; male/female, 61/10). Patients with AMI and unstable angina were included in the ACS group. Blood samples were directly collected into glass vacutainer tubes with or without EDTA disodium salt (EDTA-2Na) to obtain plasma and serum, respectively. The samples were immediately placed on ice. The anticoagulated samples were centrifuged at 1000 × *g* for 10 min, and then the plasma was carefully collected. Whole blood samples collected without EDTA-2Na were left to clot, and the serum was separated by centrifugation at 1000 × *g* for 10 min. All the samples were either subjected to measurement immediately or stored at −80 °C until measurement. All samples were analyzed in the University of Tokyo Hospital.

### Preparation of ATX-depleted serum

ATX was depleted from human serum using ATX-binding beads, as previously described^37^. Ten micrograms of biotin-anti-ATX mAb (prepared from a rat anti-ATX antibody named R10.23^38^) solution and 80 μL of Dynabeads M-280 Streptavidin suspension (Life Technologies Corp., Carlsbad, CA) were added to 500 μL of TBST for 15 min, followed by washing with TBST twice while keeping the tube on a magnet for the collecting beads. After 300 μL of serum was shaken on the magnet for 15 min, the supernatant was used as ATX-depleted serum.

### Serum ATX antigen measurement

Anti-human ATX monoclonal antibodies were produced by immunization with recombinant human ATX expressed in a baculovirus system. An automated immunoassay for the quantitative determination of ATX was then established, and human serum samples were assayed using an automated immunoassay analyzer AIA-system (TOSOH Corp., Tokyo, Japan), as previously described^38^.

### Choline determination using an enzymatic assay

Serum and plasma choline concentrations were determined using a modified enzymatic assay, as previously reported^39^. A choline molecule catalyzed by choline oxidase produces two hydrogen peroxides. In the presence of peroxidase, 4-AA and Trinder’s reagent are oxidized by the hydrogen peroxides to generate quinoneimine dye. The choline level is then determined by measuring the increased absorbance at 546 nm. Actually, 10 μL of sample was added to 180 μL of Reagent 1, containing 4-AA and peroxidase. After the incubation of this mixture for 5 min at 37 °C, 60 μL of Reagent 2, containing choline oxidase and TOOS, was added followed by another 5 min of incubation. The absorbance at 546 nm was measured at 5 and 10 min after the start of the reaction. The actual handling procedures were performed using a biochemical automatic analyzer (Hitachi 7600 [Hitachi, Tokyo, Japan] or JCA-BM8040 [JEOL, Tokyo, Japan]).

### Measurement of phospholipids and other analytes

The concentrations of sphingomyelin in the serum and of LPC and LPA in the plasma were determined using a specific enzymatic assay with sphingomyelinase^40^ and lysophospholipase^41,42^, as previously described. The total phospholipids (choline-containing phospholipids) in the samples were measured using a commercial kit (Alfresa Pharma Corp., Osaka, Japan), following the manufacturer’s protocol. We determined the serum phosphatidylcholine concentration by subtracting the sphingomyelin and LPC values from the choline-containing phospholipids, as described previously^37,40^. These phospholipid measurements were performed using an automatic analyzer (Hitachi 7600 [Hitachi, Tokyo, Japan] or JCA-BM8040 [JEOL, Tokyo, Japan]). Other ordinary markers were determined using routine laboratory methods.

### LysoPLD activity measurement

The serum lysoPLD activity was assessed by measuring choline liberation from the substrate LPC, as previously described^43^. The reactions were performed in 100 μL aliquots; the serum samples (20 μL were incubated with 2 mmol/L 1-myristoyl (14:0)-LPC (Avanti Polar Lipids Inc., Alabaster, AL, USA) in the presence of 100 mmol/L Tris-HCl, pH9.0, 500 mmol/L NaCl, 5 mmol/L MgCl_2_, 5 mmol/L CaCl_2_, and 0.05% Triton X-100 for 3 hours at 37 °C. The liberated choline was detected using an enzymatic photometric method with choline oxidase, peroxidase, and TOOS as a hydrogen donor.

### Genetically modified mice

ATX knock-out mice were prepared using the Cre-loxP system, as previously described^25^. Exons 6 and 7 in the Enpp2 gene, the active center of ATX, are flanked by loxP sites. Using this system, we prepared mice with various genetic backgrounds (wild type, lox/+, loxP/loxP, +/−, loxP/−). ATX transgenic mice (cre/+tg/+) were produced using a previously reported strategy^44^.

### Statistical analysis

The results were expressed as the mean ± SD. All data were statistically analyzed using SPSS ver. 20.0 (Chicago, IL). The Mann-Whitney test or one-way ANOVA followed by a Kruskal-Wallis post-hoc test was used to compare the choline, LPA and ATX levels between genetically modified mice and the choline levels among the NCA, SAP and ACS groups. The Spearman rank correlation test was employed to determine the significance of the correlation among various test items in healthy human subjects. A post-hoc Wilcoxon test after a Friedman test was performed to compare the choline and LPA concentrations under fasting and fed states. Demographic and clinical variables and the number of ACS patients were compared across quartiles of choline levels using the Jonckheere-Terpstra trend test and the Cochran-Armitage Test, respectively. The independent effect of the biomarkers on the risk of ACS adjusting for potential confounders was determined using a multiple logistic regression analysis. The following variables were initially incorporated into the univariable model: age, BMI, current smoking, diabetes, red blood cell count, HDL-C, LDL-C, triglyceride, hsCRP, choline quartile, and LPA tertile. Statistically significant variables in the multivariable regression analysis selected using a forward stepwise procedure were subsequently included in a new model. A *P* < 0.05 was considered statistically significant.

### Data Availability Statement

The datasets generated or analyzed during the current study are available from the corresponding author on reasonable request.

## Electronic supplementary material

Supplemental Figure(DOCX 655 kb)
