# Supplementary material for: Measurement of plasma choline in acute coronary syndrome: importance of suitable sampling conditions for this assay
Source: Sci Rep. 2018 Mar 16;8:4725. doi: 10.1038/s41598-018-23009-x (PMC5856837; doi:10.1038/s41598-018-23009-x)
Supplement: Supplementary file 1 — Supplemental Figure(DOCX 655 kb) [file 41598_2018_23009_MOESM1_ESM.docx]

**Title Page**

Supplementary Information File

Measurement of plasma choline in acute coronary syndrome: importance of suitable sampling conditions for this assay

Ryunosuke Ohkawa,^1,2^ Makoto Kurano, ^2,3^ Noboru Sakai,^4^ Tatsuya Kishimoto,^4^ Takahiro Nojiri,^2^ Koji Igarashi,^5^ Shigemi Hosogaya,^6^ Yukio Ozaki,^7^ Tomotaka Dohi,^8^ Katsumi Miyauchi,^8^ Hiroyuki Daida,^8^ Junken Aoki,^9,10^ Shigeo Okubo,^2,11^ Hitoshi Ikeda,^2,3^ Minoru Tozuka^1^ and Yutaka Yatomi^2,3,*^

^1^Analytical Laboratory Chemistry, Graduate School of Health Care Sciences, Tokyo Medical and Dental University, Tokyo, Japan

^2^Department of Clinical Laboratory, The University of Tokyo Hospital, Tokyo, Japan;

^3^Department of Clinical Laboratory Medicine, Graduate School of Medicine, The University of Tokyo, Tokyo, Japan;

^4^Diagnostics R&D Division, Alfresa Pharma Corporation, Osaka, Japan

^5^Bioscience Division, Research and Development Management Department, TOSOH Corporation, Kanagawa, Japan;

^6^Department of Medical Technology, School of Health Sciences, Tokyo University of Technology, Tokyo, Japan;

^7^Department of Clinical and Laboratory Medicine, Faculty of Medicine, University of Yamanashi, Yamanashi, Japan;

^8^Department of Cardiovascular Medicine, Juntendo University School of Medicine, Tokyo, Japan;

^9^Department of Molecular and Cellular Biochemistry, Graduate School of Pharmaceutical Sciences, Tohoku University, Miyagi, Japan;

^10^PRESTO, Japan Science and Technology Corporation, Tokyo, Japan;

^11^Department of Clinical Laboratory Medicine, Faculty of Health Science Technology, Bunkyo Gakuin University, Tokyo, Japan;

*Corresponding author: Yutaka Yatomi, MD, PhD, Department of Clinical Laboratory Medicine, Graduate School of Medicine, The University of Tokyo, 7-3-1 Hongo, Bunkyo-ku, Tokyo 113-8655, Japan

Phone: +81-3-5800-8721, Fax: +81-3-5689-0495, E-mail: [yatoyuta-tky@umin.ac.jp](mailto:yatoyuta-tky@umin.ac.jp)

Supplementary Figure Legend

Figure S1 Validation for the choline enzymatic assay. Calibration line for the choline assay (a). As standard solution, choline chloride (38.8 mmol/L) was measured. Linearity of the choline assay (b). Choline chloride was diluted with saline and measured.

Figure S1
